# CAs-Net: A Channel-Aware Speech Network for Uyghur Speech Recognition

**DOI:** 10.3390/s25123783

**Published:** 2025-06-17

**Authors:** Jiang Zhang, Miaomiao Xu, Lianghui Xu, Yajing Ma

**Affiliations:** 1 School of Computer Science and Technology, Xinjiang University, Urumqi 830017, China; zhangjiang@stu.xju.edu.cn (J.Z.); xuhui686899@gmail.com (L.X.); myj@xjnu.edu.cn (Y.M.); 2 Medical Engineering and Technology, Xinjiang Medical University, Urumqi 830017, China; 3 School of Computer Science and Technology, Xinjiang Normal University, Urumqi 830054, China

**Keywords:** low-resource speech recognition, channel modeling, multi-scale convolution

## Abstract

This paper proposes a Channel-Aware Speech Network (CAs-Net) for low-resource speech recognition tasks, aiming to improve recognition performance for languages such as Uyghur under complex noisy conditions. The proposed model consists of two key components: (1) the Channel Rotation Module (CIM), which reconstructs each frame’s channel vector into a spatial structure and applies a rotation operation to explicitly model the local structural relationships within the channel dimension, thereby enhancing the encoder’s contextual modeling capability; and (2) the Multi-Scale Depthwise Convolution Module (MSDCM), integrated within the Transformer framework, which leverages multi-branch depthwise separable convolutions and a lightweight self-attention mechanism to jointly capture multi-scale temporal patterns, thus improving the model’s perception of compact articulation and complex rhythmic structures. Experiments conducted on a real Uyghur speech recognition dataset demonstrate that CAs-Net achieves the best performance across multiple subsets, with an average Word Error Rate (WER) of 5.72%, significantly outperforming existing approaches. These results validate the robustness and effectiveness of the proposed model under low-resource and noisy conditions.

## 1. Introduction

Automatic Speech Recognition (ASR) aims to convert human speech into text information that can be easily understood and processed by machines, thereby supporting downstream tasks such as semantic understanding, command execution, or information retrieval [1]. As a result, ASR has become a core technology in modern human–computer interaction systems, with wide applications in virtual assistants, in-vehicle voice control, and accessible communication. With the rapid development of deep learning, particularly the emergence of end-to-end neural network models, ASR systems have achieved remarkable improvements in recognition accuracy and robustness. However, current ASR systems still face significant challenges in modeling the complex temporal dependencies in speech signals and in capturing fine-grained structural information.

Traditional speech recognition models, such as RNN-Transducer [2], CTC-based models [3], and attention-based encoder–decoder architectures [4], primarily focus on temporal alignment between frames. These models typically treat the encoder outputs as sequences of high-dimensional vectors, each corresponding to a frame, and often process them under the assumption that all feature dimensions (channels) are independent and equally important. However, this global representation may overlook the local structural dependencies among channels within each frame, which are essential for capturing fine-grained patterns in speech.

To address this limitation, channel modeling has emerged as a promising direction for enhancing the internal representation of speech features. Instead of treating all channels equally, channel modeling aims to capture the interactions and dependencies across different feature dimensions within a frame. By doing so, it can uncover complementary or redundant information among channels, leading to more informative and discriminative feature representations. Nevertheless, existing approaches often rely on fixed convolutional kernels or generic attention mechanisms, which may struggle to adaptively capture the dynamic and structured relationships across channels. This underscores the need for a more flexible and expressive channel modeling mechanism tailored to the complex nature of speech signals.

Furthermore, most existing ASR research has focused on high-resource languages and large-scale datasets, where abundant training data can compensate for model limitations. In contrast, low-resource languages—especially under noisy conditions—pose greater challenges due to limited data availability and increased variability. These scenarios demand not only data-efficient training strategies but also robust architectural designs that can generalize well under constrained conditions.

To better model the structural information within the channel dimension of the Conformer encoder output, we propose a novel feature transformation strategy called Channel Rotation. Specifically, the one-dimensional channel vector of each frame (e.g., 256 dimensions) is reshaped into a two-dimensional matrix (e.g., 16 × 16) and subjected to a rotation operation. This spatial manipulation primarily captures the local structural relationships within the channel dimension and does not directly involve modeling inter-frame dependencies. Compared with traditional convolutional neural networks (CNNs) [5,6], our Channel Rotation operation offers a significantly larger local receptive field within the channel dimension, enabling the model to perceive richer channel-wise information with fewer layers, whereas CNNs are constrained by the slow expansion of the receptive field. By subsequently integrating the powerful global modeling capabilities of the Transformer architecture, our approach effectively combines both local and global information modeling, thereby enhancing the model’s ability to capture long-range temporal dependencies. Moreover, considering the challenges posed by low-resource languages like Uyghur—such as limited training data, rich phonetic variations, and imbalanced phoneme distributions—we further incorporate a multi-scale convolutional module into the model architecture. This module improves the model’s capacity to perceive features at various temporal scales, allowing it to effectively capture both short- and long-term acoustic patterns. Combined with the Channel Rotation mechanism, this design significantly boosts the model’s ability to recognize fine-grained speech units in Uyghur, maintaining strong performance even in noisy environments.

The main contributions of this paper are as follows:

1. We propose a Channel Rotation Module that reconstructs each frame’s channel vector into a spatial structure and applies a rotation operation to explicitly model the local structural relationships within the channel dimension. This addresses the structural limitations of conventional ASR encoders, which often ignore internal channel interactions, thereby enhancing the model’s contextual representation capabilities.
2. We design a multi-scale convolutional structure within the Transformer framework to better accommodate the linguistic characteristics of low-resource languages such as Uyghur, which often exhibit compact articulation and complex temporal rhythms. This design improves the model’s ability to perceive and abstract multi-scale temporal information.
3. Extensive experiments are conducted on multiple benchmark speech recognition datasets. Results demonstrate that the proposed method achieves significant improvements in recognition accuracy and robustness, particularly under low-resource and noisy acoustic conditions.

## 2. Related Work

### 2.1. Classical and Neural Network-Based Speech Recognition Models

Traditional speech recognition systems predominantly relied on the Hidden Markov Model (HMM), which models the temporal dynamics of speech as transitions between hidden states, each generating observable acoustic features [7]. HMMs were often coupled with Gaussian Mixture Models (GMMs) to estimate state emission probabilities [8]. While this architecture enabled early success, its assumptions—such as frame-level independence and limited modeling of nonlinearity—restricted its capacity to handle long-term dependencies, noise, and low-resource scenarios.

To overcome these limitations, Deep Neural Networks (DNNs) were introduced to serve as powerful acoustic models [9,10]. DNNs learn hierarchical features and can model complex, nonlinear mappings from acoustic features to linguistic units. Nevertheless, DNNs still face challenges in noisy or low-resource environments due to their reliance on large-scale supervised training data [11].

Convolutional neural networks (CNNs) further advanced speech recognition by introducing inductive biases such as locality and weight sharing, making them more robust to spectral variations and background noise [6,12]. CNNs are particularly effective at modeling local acoustic patterns, such as formant transitions or phoneme boundaries. They are now widely used in end-to-end ASR pipelines as front-end feature encoders.

However, CNN-based methods implicitly model local channel patterns through fixed convolutional kernels, which limits their flexibility in adapting to cross-channel dependencies or complex local–global interactions. Moreover, CNNs often struggle to capture global temporal dependencies and cannot inherently process hierarchical or multi-scale speech characteristics—issues particularly evident in complex, low-resource languages such as Uyghur, which exhibit rich prosodic and syllabic diversity [13,14,15].

To address these gaps, multi-scale convolutional modeling has been explored, using varying kernel sizes to better extract features at different temporal resolutions. Our work builds upon this by integrating both multi-scale receptive fields and channel-structural rotation to model fine-grained intra-frame dependencies—key to capturing the phonological complexity of Uyghur speech.

### 2.2. Sequence Modeling and Global Contextual Representation

To capture long-term temporal dependencies, researchers introduced Recurrent Neural Networks (RNNs) and their gated variants—Long Short-Term Memory (LSTM) and Gated Recurrent Unit (GRU)—which mitigate vanishing gradients through gating mechanisms [16,17]. These autoregressive models excel in sequential modeling, especially in sequence-to-sequence (Seq2Seq) architectures but are inherently limited in parallelization and training efficiency [18,19].

The introduction of the Transformer model, built on self-attention mechanisms, revolutionized speech recognition by enabling the direct modeling of global dependencies with parallel computation [20,21]. Transformers allow the model to dynamically attend to relevant parts of the input sequence, thereby overcoming limitations of recurrent architectures. They form the backbone of many state-of-the-art ASR systems.

However, Transformer-based models typically treat channel tokens equally and overlook structured relationships across feature channels. This uniform treatment neglects local priors that are crucial for capturing phonetic cues, particularly in languages with intricate local structures. While the Conformer model [22] attempts to incorporate both convolution and attention, its design still lacks the mechanisms to explicitly disentangle and reconstruct structured channel-wise relationships within frames.

To address this, we propose a Channel Rotation module that reorganizes and re-encodes feature channels through spatial rotation strategies. This enables the model to jointly model local channel-wise structures and global contextual interactions, offering enhanced modeling capacity for languages like Uyghur and improving robustness under noisy conditions.

### 2.3. Channel Modeling in Speech Recognition

Channel modeling remains an underexplored yet fundamental aspect of speech representation learning. Prior works have attempted to improve channel utilization through Squeeze-and-Excitation (SE) blocks and Channel Attention mechanisms [23,24,25,26]. These approaches dynamically reweight channel responses based on global statistics, enhancing the model’s sensitivity to informative features. While effective, they operate in a global pooling context and lack the ability to model explicit structural transformations across channels.

In contrast, our method goes beyond reweighting: we propose a spatial channel reconstruction and rotation strategy that treats the channel dimension as a meaningful spatial axis, enabling structured transformations and enhanced local–global interaction. This approach can be viewed as a form of dynamic spatial reasoning within the feature channel space, offering a novel pathway to exploit channel structure beyond traditional attention or excitation mechanisms.

### 2.4. Large-Scale Models and Low-Resource Language Limitations

Recently, the Whisper model has emerged as a large-scale, end-to-end speech recognition framework based on a Transformer encoder–decoder architecture [27]. Trained on 680,000 h of multilingual and multitask data, Whisper demonstrates strong robustness to noise and cross-lingual generalization. It models speech via log-Mel spectrogram inputs and performs multilingual ASR and translation through task-specific tokens.

However, Whisper’s architectural scale and training objectives are not optimized for low-resource or structurally complex languages such as Uyghur. Its monolithic design lacks the mechanisms to dynamically adapt to language-specific structural priors, and its parameter count renders it unsuitable for constrained environments. Our method offers a more compact and language-aware solution, emphasizing the role of channel structural modeling to better support speech recognition in underrepresented languages.

## 3. Methodology

**Figure 1:**
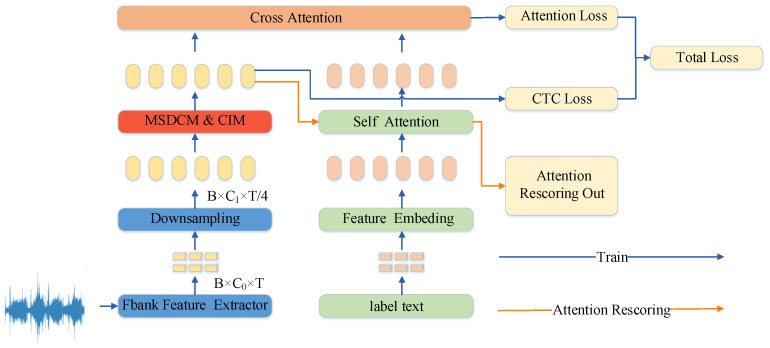
Overall architecture figure of CAs-Net.

This paper proposes a robust speech recognition model tailored for the Uyghur language, termed the Channel-Aware Speech Network (CAs-Net), designed to address the dual challenges of data scarcity and environmental noise in speech recognition. The architecture integrates multi-scale feature modeling, a channel interaction mechanism, and a U2-based decoding strategy, leading to significant improvements in the recognition performance under complex linguistic structures and noisy acoustic conditions. The overall framework of the proposed model is illustrated in Figure 1.

As illustrated in Figure 1, the raw speech signal is first transformed into Mel-filterbank (Fbank) features, which serve as the input representation to the model. These features are then passed through a downsampling module to reduce the dimensionality while preserving essential temporal information. Subsequently, the feature sequence is fed into the Multi-Scale Depthwise Convolution Module (MSDCM). This module integrates pointwise convolutions, multi-scale depthwise separable convolutions, and a self-attention mechanism, enabling it to capture both fine-grained local variations and long-range dependencies. The multi-scale design enhances adaptability to varying speech rates and pronunciation styles, while the self-attention mechanism strengthens the model’s focus on critical information, thereby improving the overall acoustic modeling performance.

### 3.1. Multi-Scale Depthwise Convolution Module

**Figure 2:**
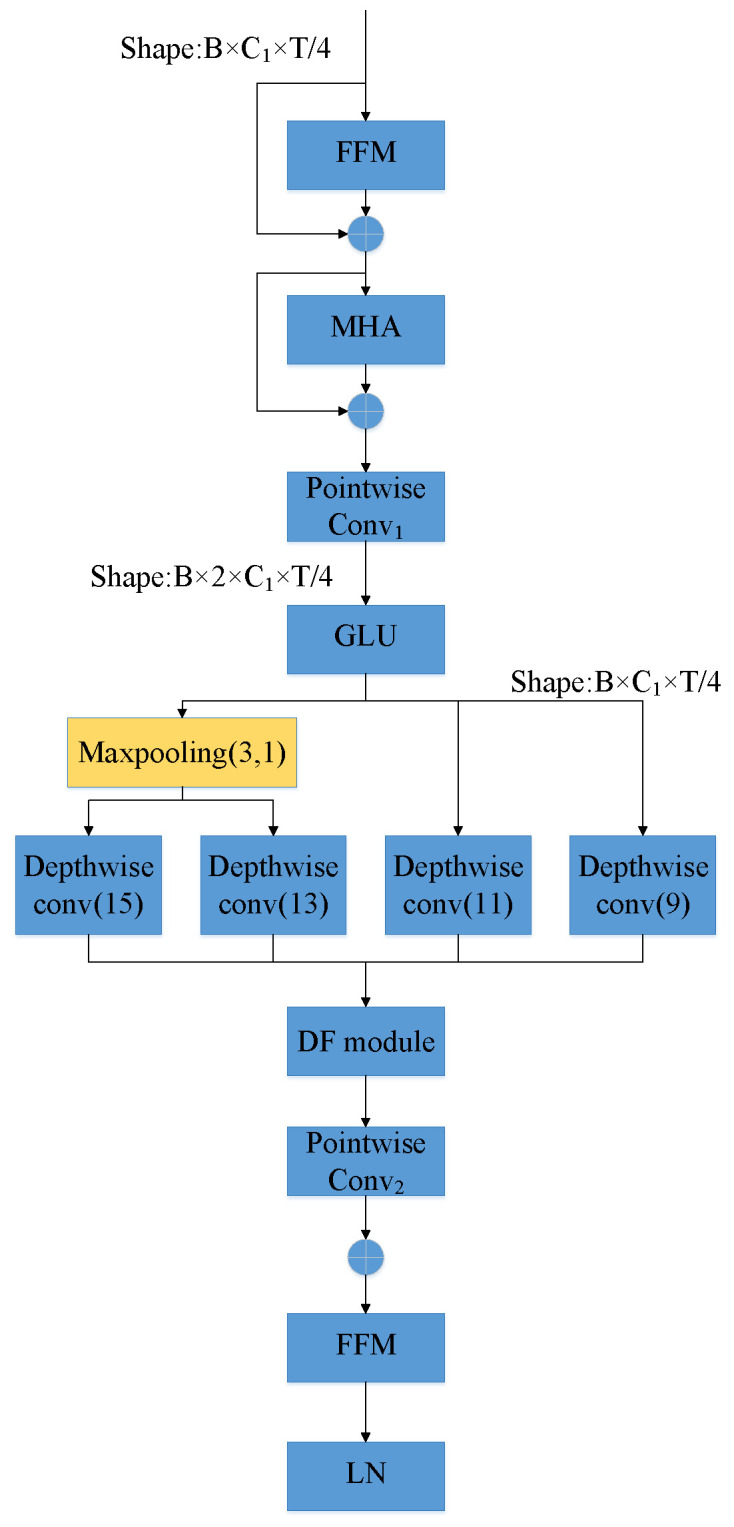
MSDCM architecture figure.

To enhance the modeling capability of speech recognition systems in low-resource language scenarios—especially in adapting to variations in speaking rate and contextual conditions—we propose a Multi-Scale Depthwise Convolution Module (MSDCM). This module integrates multi-scale depthwise separable convolutions with a self-attention mechanism, enabling it to capture local dynamic patterns while incorporating global contextual information. As a result, it significantly improves the diversity and robustness of the feature representations. The architecture of the module is illustrated in Figure 2.

As illustrated in the figure, the overall architecture of the MSDCM consists of four core components: pointwise convolution, multi-scale depthwise separable convolution, self-attention, and residual connection with layer normalization. The input features are first processed by a 1×1 pointwise convolution to adjust the channel dimension without altering the temporal resolution, thereby providing a unified representation for subsequent operations. To capture contextual information across different temporal ranges, the module employs several parallel depthwise separable convolution branches with varying kernel sizes (9, 11, 13, and 15). Each branch comprises a depthwise convolution to model temporal structures and a pointwise convolution for channel-wise fusion. For branches with larger kernel sizes (e.g., 13 and 15), a max-pooling operation is applied before the depthwise convolution to reduce the temporal resolution and computational cost, while also enhancing the module’s ability to abstract higher-level features from broader contexts. The outputs of all branches are concatenated along the channel axis and further integrated via another 1×1 convolution. To enhance the modeling of global temporal dependencies, a lightweight self-attention mechanism is incorporated, enabling the module to extract relationships among key time steps and compensate for the limited receptive field of convolutional operations. Finally, residual connections and layer normalization are applied to stabilize training and mitigate the vanishing gradient problem in deeper networks.

(1) $$\begin{array}{cc} X_{out}= & W*\mathrm{Activation}(\mathrm{Norm}(\alpha_{1}X_{9}+\alpha_{2}X_{11}\\ & +\alpha_{3}\left(\mathrm{Maxpooling}\left(X_{13}\right)\right)\\ & +\alpha_{4}\left(\mathrm{Maxpooling}\left(X_{15}\right)\right)))+b. \end{array}$$

The final output of the module is represented by Equation (1), where *X* is the input to the module, ${\mathrm{Conv}}_{\mathrm{multi-scale}}$ represents a deep convolutional structure with multi-scale branches, and Attention denotes a lightweight self-attention mechanism. This module simultaneously models local context while incorporating global dependency modeling, effectively enhancing the model’s robustness in varying speech rates, complex linguistic structures, and noisy environments.

### 3.2. Channel Interaction Module

Although multi-scale convolutions and self-attention mechanisms provide strong modeling capabilities along the temporal dimension and offer a certain degree of channel interaction, they remain limited in their ability to capture inter-channel dependencies due to restricted receptive fields. In speech representation, the channel dimension often contains rich semantic and structural information. This is particularly crucial in low-resource languages such as Uyghur, where different channels may correspond to distinct frequency bands or abstract phonetic patterns. Fully modeling the relationships between channels can significantly enhance the model’s ability to capture key speech cues and improve recognition performance. To this end, we propose a Channel Interaction Module (CIM), which explicitly models the dependencies among channels and strengthens inter-channel information fusion, thereby improving the expressiveness and robustness of the acoustic model.

The Channel Interaction Module (CIM) is designed to effectively model inter-channel correlations. Its core idea is to reshape and reorganize the channel features to expose their spatial relationships, then enhance these relationships by rotating the spatial layout to simulate diverse dependency patterns. In our model, the input acoustic features undergo a 4× temporal downsampling, resulting in an intermediate representation of shape $R^{B\times C\times T/4}$, where *B* denotes the batch size, *T* is the original frame length, and *C* is the channel dimension, typically set to 256. To better capture inter-channel dependencies, we focus on the channel-wise structure of each time step. For a given time frame, the channel vector $x\in R^{C}$ is reshaped into a two-dimensional channel map:

(2) $$x\in R^{C},\;\mathrm{with}\;C=256$$

(3) $$X_{0}=\mathrm{Reshape}\left(x\right)\in R^{H\times W},\;\mathrm{where}\;H=W=16$$

**Figure 3:**
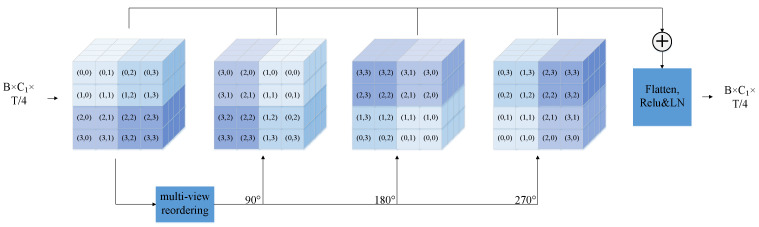
CIM architecture figure.

This transformation converts the original 1D semantic vector into a 2D spatial layout, allowing the model to perceive and exploit local spatial patterns among channels. In practice, this layout preserves the local adjacency of channels, enabling subsequent operations (e.g., rotation) to model spatial correlations more effectively. For visualization clarity, Figure 3 illustrates a 4×4 example instead of the full 16×16 layout.

To simulate diverse spatial dependencies across channels, we apply a sequence of index-based reordering and transposition operations on the reshaped 2D channel maps. Specifically, given a channel map $X_{0}\in R^{H\times W}$, we apply a series of gather operations with a pre-defined index tensor, followed by spatial transpositions using Permute(H,W). This iterative process generates multiple views $X_{0},O_{1},O_{2},O_{3}$, each corresponding to a distinct reordering pattern that simulates a rotated perspective of the channel features:

(4) $$\begin{array}{cc} O_{1} & =\mathrm{Permute}\left(\mathrm{Gather}\left(X_{0},\mathrm{index}\right),\left(H,W\right)\right),\\ O_{2} & =\mathrm{Permute}\left(\mathrm{Gather}\left(O_{1},\mathrm{index}\right),\left(H,W\right)\right),\\ O_{3} & =\mathrm{Permute}\left(\mathrm{Gather}\left(O_{2},\mathrm{index}\right),\left(H,W\right)\right) \end{array}$$

These multi-angle views are then fused via convolutional or linear layers to integrate information across rotated versions, resulting in a richer and more diverse representation that captures both local and global channel dependencies. This design substantially enhances the capacity of the model to capture key acoustic cues, especially under noisy conditions or in low-resource scenarios such as Uyghur. By explicitly modeling and augmenting the inter-channel structure, CIM significantly contributes to improved recognition robustness and accuracy.

To achieve this, we perform a weighted fusion of the original and rotated channel maps, followed by a nonlinear transformation and residual connection. Specifically, the fusion and final output are computed as

(5) $$\begin{array}{cc} X_{\mathrm{fused}} & ={\mathrm{ReLU}}_{1}\left(0.5\cdot \left(O_{1}+O_{2}+O_{3}\right)+0.5\cdot X_{0}\right)\\ X\mathrm{out} & =\mathrm{ReLU}2\left(\mathrm{LayerNorm}\left(\mathrm{Flatten}(X_{\mathrm{fused}})\right)+X_{\mathrm{skip}}\right) \end{array}$$

Here, $X_{0}$ is the original channel map; $O_{1}$, $O_{2}$, and $O_{3}$ are its rotated versions; and $X_{skip}$ is the residual input. LayerNorm ensures stable activation statistics before the residual fusion.

## 4. Experiment and Analysis

### 4.1. Datasets

In our experiments, we employed multiple types of data, including standard open-source speech datasets and a small-scale self-constructed dataset. For the open-source datasets, we used the Uyghur language subsets of the CommonVoice corpus, specifically versions 7, 8, 9, and 16, denoted as Ug 7, Ug 8, Ug 9, and Ug 16, respectively [28]. These subsets were collected through Mozilla’s web-based crowdsourcing platform, where volunteers contributed recordings by reading pre-defined prompts. Each utterance is associated with a corresponding transcription, and many recordings are validated by independent annotators to ensure data quality. The speaker population covers a wide range of ages, genders, and regional accents, which contributes to the dataset’s linguistic diversity.

The recordings were made using various devices in uncontrolled environments, including quiet indoor settings as well as everyday background conditions, thus providing a realistic distribution of speech quality. The total durations of Ug 7, Ug 8, Ug 9, and Ug 16 were 44, 64, 65, and 180 h, respectively. However, to ensure consistency and reliability, we conducted a data cleaning process that removed low-quality, unverified, or mismatched samples based on alignment accuracy, duration thresholds, and audio availability. After this preprocessing, the resulting usable durations were 42, 60, 63, and 147 h, respectively. These cleaned subsets served as the core training and evaluation data in our experiments.

Although the total amount of data may appear relatively large at first glance, Uyghur is still widely regarded as a low-resource language in the field of speech recognition. This classification stems from the lack of large-scale curated corpora, the limited availability of high-quality transcribed speech, and the absence of mature commercial or industrial-grade ASR systems. Most publicly available Uyghur speech datasets are relatively small in scale and often contain noisy, unverified, or inconsistent segments. In addition, the number of researchers and dedicated projects focusing on Uyghur ASR remains limited, which further slows down the development of linguistic resources and technological advancement for the language. Recent work by Lu et al. (2025) also reaffirms this categorization, emphasizing that Uyghur remains underrepresented in large-scale language model training and requires dedicated strategies to enhance its translation and representation capabilities within multilingual systems [29]. In this context, the datasets used in our study—though moderate in absolute size—remain consistent with the low-resource conditions commonly encountered in Uyghur ASR research.

To simulate realistic noisy environments, we constructed synthetic noisy speech datasets by adding environmental noise to the clean speech data. The noise samples were selected from the DEMAND (Diverse Environments Multichannel Acoustic Noise Database), focusing on two typical urban transportation scenarios: BUS and METRO [30]. BUS noise was recorded inside operational public buses in downtown Vancouver, containing a mixture of engine sounds, wheel–road friction, vehicle vibrations, passenger conversations, and door movements—characterized by non-stationary and diverse interferences. METRO noise was recorded in subway systems and includes high-intensity continuous rumble from high-speed train movement, track friction, onboard announcements, and ambient station noise—representing highly complex and dynamic acoustic conditions. Both types of noise were originally recorded using the Microcone microphone array at a sampling rate of 48 kHz, providing multichannel recordings. In our experiments, we uniformly used the single-channel version of each noise recording and downsampled them to 16 kHz to better reflect the real-world deployment conditions of speech recognition systems.

To simulate realistic noisy environments, we added various types of noise to clean speech. The process involves computing the energy of both the speech and noise signals, scaling the noise according to a target signal-to-noise ratio (SNR), and then mixing it with the speech. To enhance variability, the actual SNR values follow a standard normal distribution centered at 0 dB, introducing randomness—68.27% of samples fall within [−1, 1] dB. Noise types are selected using a Dirichlet distribution to ensure diverse and unpredictable background conditions.

In addition to the use of standard public datasets, this section also incorporates a self-constructed Uyghur speech dataset to evaluate the model’s performance under real-world noisy conditions. The dataset has a total duration of approximately 5 h and is divided into two subsets, labeled Real1 and Real2.

Real1 was recorded in noisy public environments characterized by diverse and unstructured background sounds, such as overlapping human conversations and object collisions. Real2, on the other hand, was collected inside public vehicles, capturing a variety of acoustic interferences, including onboard speech, station announcements, and vehicle horns. Both subsets were recorded under natural conditions without artificial noise control, making them highly representative of practical deployment scenarios.

The recordings were produced by native Uyghur speakers of varying ages and genders to ensure speaker diversity and natural pronunciation patterns. This helps simulate realistic user scenarios and supports the development of more robust and inclusive recognition systems.

This customized dataset provides a focused and realistic benchmark for assessing the robustness of Uyghur speech recognition models in acoustically challenging environments. Experimental evaluations on Real1 and Real2 further demonstrate the proposed model’s robustness and adaptability in real-world noise conditions, offering strong support for its practical applicability.

### 4.2. Experimental Setup

In Automatic Speech Recognition (ASR) tasks, commonly used evaluation metrics include Character Error Rate (CER) and Word Error Rate (WER). For agglutinative languages such as Uyghur, which use words as the basic semantic units, employ whitespace-based tokenization, and exhibit rich morphological variations, WER is generally considered the more representative and informative metric. The WER is calculated as follows:

(6) $$\mathrm{WER}=\frac{S+D+I}{N}$$

Here, *S* denotes the number of substitutions, *D* denotes deletions, *I* denotes insertions, and *N* is the total number of words in the reference transcription. It is important to note that due to the presence of insertion errors, the number of words in the recognized output may exceed that in the reference, resulting in a WER exceeding 100%. This metric provides a comprehensive evaluation of word-level recognition accuracy and is particularly suitable for languages like Uyghur, where words serve as the fundamental semantic units.

During the construction of the synthetic noisy dataset, we incorporated a Signal-to-Noise Ratio (SNR) control mechanism by adjusting the energy ratio between the noise and the original clean speech. Since the raw noise signals typically exhibit lower energy, directly mixing them with speech would result in insufficient acoustic interference. To address this, we applied gain amplification to the noise signals before mixing, ensuring that the final SNR remains at a relatively low level. This strategy allowed us to better simulate challenging real-world conditions for evaluating speech recognition robustness.

Table 1 summarizes the main hyperparameter settings used for training the speech recognition models in this study. The encoder and decoder consist of 12 and 6 layers, respectively, with a feed-forward network dimension (Dff) of 2048 to enhance the model’s representation capacity. The number of training epochs is set to 140, with an initial learning rate of 0.0005. A gradient accumulation strategy (Accum_Grad = 4) is employed to support a larger effective batch size. For acoustic features, 80-dimensional Mel-frequency cepstral coefficients (MFCCs) are extracted (Num_Mel = 80) with a frame length of 25 ms and frame shift of 10 ms. Spectral augmentation (Spec_Aug = True) is enabled to improve robustness in noisy environments. Model training is conducted on a server equipped with four NVIDIA Tesla T4 GPUs, an Intel Xeon Gold 5320 CPU, and 257 GB of RAM, providing sufficient resources for large-scale training. The software environment is based on Python and PyTorch, with key dependencies including PyTorch 1.13.0, Torchaudio 0.13.0, and SentencePiece. The training process is visualized and monitored using TensorBoard.

### 4.3. Comparative Experiments

In this study, we perform comparative evaluations on a standard noise-free dataset using seven representative speech recognition models to comprehensively assess the performance of our proposed approach.

To begin with, the Transformer (Wenet) [31] serves as a typical Attention-based Encoder–Decoder (AED) framework, representing an early generation of end-to-end speech recognition models grounded in pure attention mechanisms. The Conformer model [22], selected as our baseline, effectively integrates convolutional layers and self-attention to capture both local and global dependencies, yielding strong performance across various tasks. Conformer_bi [31] extends the original Conformer by incorporating a bidirectional decoding structure, which enhances the model’s ability to utilize contextual information. Squeezeformer [32] adopts a Temporal U-Net style encoder that significantly reduces model complexity while maintaining competitive accuracy, making it well-suited for deployment in low-resource scenarios. Squeezeformer_bi [33] further integrates a bidirectional decoder into the Squeezeformer framework to improve sequence modeling. Efficient Conformer V1 [34] reduces computational cost through the use of grouped attention and introduces a stride-based mechanism to accelerate training. Building on this, Efficient Conformer V2 further refines the architecture by adjusting the number of attention groups and optimizing the stride settings to better balance performance and efficiency. Lastly, Paraformer (U2) [35] is a non-autoregressive end-to-end model designed for fast inference. By enabling parallel prediction, it provides a favorable trade-off between recognition speed and accuracy, particularly beneficial for real-time applications.

As shown in Table 2, the proposed CAs-Net achieves relatively low recognition error rates across four standard noise-free test sets (Ug 7, Ug 8, Ug 9, and Ug 16). Compared with the baseline model Conformer, CAs-Net achieves relative error rate reductions of 6.08, 4.22, 5.60, and 0.36 percentage points on Ug 7, Ug 8, Ug 9, and Ug 16, respectively, with the most notable improvement observed on Ug 7. Compared to the Conformer_bi model, the proposed CAs-Net achieves error rate reductions of 2.64, 2.59, 5.92, and 1.95 percentage points on the same test sets, showing a significant performance gain, especially on Ug 9. When compared with the E_ConforV2 model, CAs-Net demonstrates relative error rate reductions (or increases) of 3.53, 7.59, 5.20, and 0.56 percentage points on Ug 7, Ug 8, Ug 9, and Ug 16, respectively, with particularly notable improvements on Ug 8 and Ug 9.

The performance improvement of the proposed model can be mainly attributed to two key components. First, the relevance-aware attention mechanism enhances the model’s ability to capture fine-grained relationships between encoder outputs and decoder states. Second, the hybrid decoding structure improves context integration by leveraging the advantages of different decoding paths. These two modules significantly strengthen the model’s temporal modeling capability and decoding accuracy while maintaining low model complexity. Compared with the conventional Transformer, our model performs particularly well on Ug 7, which can be attributed to the Transformer’s limited capacity for fine-grained feature modeling. Although the Conformer and its variants show relatively strong performance on Ug 8 and Ug 9, they still underperform on Ug 7 compared to our model. This indicates that the proposed model’s strength in fine-grained feature modeling contributes to its superior performance across multiple Uyghur speech datasets.

To evaluate the robustness and recognition accuracy of the proposed model under noisy conditions, this section conducts a series of comparative experiments. Based on the Ug dataset, multiple synthetic noisy datasets are constructed by adding various types of noise. Each noisy dataset is configured with an SNR of −6 dB, simulating real-world interference. The test data are also corrupted with noise at the same SNR to ensure consistency between experimental conditions and practical application environments. This setup enables a systematic evaluation of the model’s performance under different noise conditions and validates its robustness in synthetic noisy environments. The results are summarized in Table 3, where four decoding metrics are reported—A (attention), Ar (attention-rescoring), CTC, and AV (average of A, Ar, and CTC)—all in terms of WER. These metrics jointly demonstrate the performance and robustness of the model under different decoding strategies.

Overall, CAs-Net demonstrates strong robustness on synthetic noise datasets, particularly under high noise interference conditions, where its recognition performance on several standard test sets outperforms current state-of-the-art models. However, it is important to note that while synthetic noise can simulate certain types of interference found in real-world scenarios, its noise characteristics and distribution still do not fully replicate the complexities of noise in actual environments. To further assess the model’s robustness in practical applications, this section evaluates each model using noise data collected from real-world environments. The specific results are shown in Table 4, and by comparing the recognition performance under real noise conditions, a more comprehensive assessment of each model’s adaptability in complex real-world scenarios can be made.

As shown in Table 4, we first synthesize the standard test sets, Ug 7, Ug 9, and Ug 16, with BUS noise at a −6 dB SNR. The model is then trained using these noisy training sets. Subsequently, the trained model is evaluated on two real-world noise environment datasets, Real1 and Real2, to assess its recognition performance in practical settings.

The results in Table 4 demonstrate that the proposed CAs-Net model achieves the lowest error rates across all test data and both real-world environments. For instance, in the Real1 environment, CAs-Net achieves error rates of 5.6, 2.6, and 2.5 on Ug 7, Ug 9, and Ug 16, respectively, significantly outperforming other state-of-the-art models such as Conformer (17.6, 6.3, 5.9) and Efficient Conformer V2 (6.5, 4.2, 6.7). Notably, on Ug 7, it improves by 12.0 percentage points compared to Conformer and 0.9 percentage points compared to Efficient Conformer V2. In the Real2 environment, despite more complex noise levels and speaker conditions, CAs-Net demonstrates strong robustness, achieving error rates of 10.7, 7.0, and 5.9 on Ug 7, Ug 9, and Ug 16, respectively, still outperforming other methods. Particularly, on Ug 9, it reduces the error rate by 24.8 percentage points compared to Conformer (31.8) and by 28.2 percentage points compared to Conformer_bidecoder (35.2), showcasing its strong adaptability.

To validate the statistical significance of performance improvement, we conduct a paired *t*-test on the full Ug7 dataset under −6 dB SNR conditions. The resulting *p*-value is $5.9482\times 10^{-7}$, indicating a highly significant difference between the proposed method and the baseline.

To further assess robustness, we randomly sample 100 utterances from the test set and repeat the evaluation 10 times. As shown in Table 5, most of the random samples also yield *p*-values below the conventional significance threshold of 0.05, supporting the stability and reliability of the observed improvements.

### 4.4. Ablation Study

To systematically verify the contributions of the proposed Channel Interaction Module (CIM) and Multi-Scale Depthwise Convolution Module (MSDCM), we conduct a series of ablation experiments under various noise types and SNR conditions. These experiments are designed not only to observe performance changes but also to theoretically validate the role of each component in improving noise robustness.

Specifically, the CIM module enhances the encoder’s ability to capture structured dependencies within the channel dimension. By reconstructing the channel-wise vector of each frame into a 2D spatial map and applying permutation-based rotations, CIM simulates different perspectives of local channel interactions. This design addresses a common limitation in conventional ASR encoders, which often treat channels independently and overlook their internal structural relationships. In the ablation setup, we define two simplified variants: -O3, which disables the third rotation operation, and -O2-O3, which disables both the second and third rotations. This allows us to evaluate how much each view contributes to the final performance.

On the other hand, the MSDCM module is tailored to capture temporal information at multiple scales. This is particularly beneficial for low-resource languages such as Uyghur, which exhibit compact phonetic articulation and non-uniform temporal dynamics. By embedding multi-scale depthwise convolutions into the Transformer architecture, the model can better learn both short-term and long-term dependencies, enhancing its temporal abstraction ability.

The results, summarized in Table 6 and Table 7, show that removing either CIM or MSDCM leads to consistent degradation in recognition accuracy across all tested noise conditions. This supports our theoretical claim that both modules contribute complementary strengths—CIM for structural channel modeling and MSDCM for temporal scalability—ultimately resulting in enhanced robustness and improved speech recognition performance under adverse acoustic conditions.

### 4.5. Visualization

**Figure 4:**
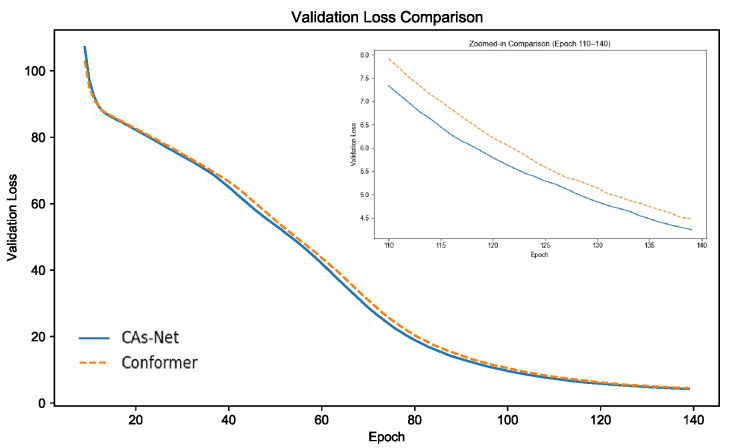
Validation loss comparison between CAs-Net and the Conformer.

To further analyze the optimization behavior of the proposed model during training, we plot the validation loss curve, as shown in Figure 4. The left part of the figure illustrates the overall trend of validation loss throughout the training process. It can be observed that the proposed CAs-Net model exhibits a rapid decline in loss within the first 30 training batches, significantly outperforming the baseline Conformer model. This indicates that our model achieves faster convergence and demonstrates higher training efficiency. The right part of the figure presents a magnified view of the validation loss between steps 110 and 140, highlighting the detailed performance differences between the two models. As shown, CAs-Net consistently maintains a lower loss compared to the baseline during this stage, validating its advantages in terms of stability and convergence quality.

These results benefit from the incorporation of two key architectural components in our model: On the one hand, the Channel Interaction Module (CIM) explicitly models high-order dependencies among channels through spatial structuring and rotation-enhanced operations, thereby improving the expressive power of feature representations and enabling the model to more efficiently and accurately capture critical information. On the other hand, the Multi-Scale Depthwise Convolution Module (MSDCM) combines multi-branch depthwise convolutions with a lightweight self-attention mechanism, enhancing the model’s ability to perceive linguistic patterns at different temporal scales. This further improves the model’s learning efficiency for complex structures. As a result, CAs-Net achieves faster loss reduction during both early and mid-training stages, demonstrating superior optimization performance and robustness.

**Figure 5:**
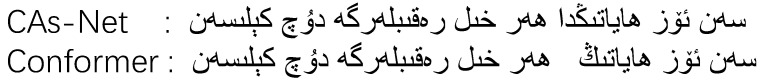
Recognition output comparison between Conformer and CAs-Net.

A qualitative analysis of recognition errors is presented in Figure 5, comparing the proposed CAs-Net with the Conformer baseline on identical speech inputs. As illustrated, CAs-Net produces a fully correct transcription, while Conformer makes recognition errors. Although both outputs preserve the general semantic content, Conformer introduces inaccuracies at the lexical level, which could potentially affect understanding in downstream tasks. Specifically, Conformer omits a crucial suffix equivalent to “in” in English, leading to an incomplete phrase. For reference, the recognition results are translated as follows: CAs-Net’s output is “You will encounter various opponents in your life,” while Conformer’s output is “You your life will encounter various opponents.” This example highlights the advantage of CAs-Net in capturing fine-grained details, especially in scenarios requiring high transcription fidelity.

## 5. Conclusions

This paper introduces CAs-Net, an end-to-end speech recognition model tailored for low-resource languages. Through explicit channel interaction and multi-scale temporal modeling, the model effectively enhances structural representation and contextual awareness, leading to robust performance across a range of acoustic conditions.

Extensive experiments on Uyghur speech demonstrate that CAs-Net achieves notable improvements over baseline models, with an average WER of 5.72% even under real-world noise. These results suggest the potential applicability of the proposed framework to other agglutinative or structurally similar low-resource languages.

While the current work focuses on Uyghur, future research will explore broader cross-lingual generalization, further efficiency optimization, and the integration of self-supervised techniques to better leverage unlabeled data.

## Figures and Tables

**Table 1:** Experimental parameter settings.

Parameter Name	Parameter Value
Num_Emcoder	12
Num_Decoder	6
Dff	2048
Epoch	140
Lr	0.0005
Num_Mel	80
Frame_Length	25
Frame_Shift	10
Spec_Aug	True
Accum_Grad	4

**Table 2:** Performance comparison on standard noise-free Uyghur speech datasets.

Model	WER (↓)			
	Ug 7	Ug 8	Ug 9	Ug 16
Transformer(Wenet) [31]	20.87	15.02	14.07	6.28
Conformer [22]	11.83	8.27	7.53	5.60
Conformer_bi [31]	8.39	6.64	7.85	7.19
Squeezeformer [32]	11.67	9.67	15.50	5.89
Squeezeformer_bi [33]	11.61	9.23	10.75	6.76
E_ConforV1 [34]	8.56	12.51	8.98	5.90
E_ConforV2 [34]	9.28	11.64	7.13	4.68
Paraformer (U2) [35]	14.64	-	-	6.15
CAs-Net	5.75	4.05	1.93	5.24

**Table 3:** Comparison experiment at SNR = −6.

Noise	Model	Ug 7				Ug 16			
		A	Ar	CTC	AV	A	Ar	CTC	AV
BUS (−6 dB)	Squeezeformer	17.2	53.2	61.6	44.0	4.4	16.0	23.3	14.5
	Squeezeformer_bi	63.3	58.7	65.1	62.4	6.2	15.0	21.4	14.2
	Transformer	104.2	97.0	97.4	99.5	20.7	52.1	59.7	44.2
	EfficonformerV2	7.7	20.3	29.4	19.1	6.0	12.1	17.0	11.7
	EfficonformerV1	6.7	23.0	32.5	20.8	6.2	12.5	17.5	12.1
	Conformer	2.4	13.0	20.0	11.8	6.0	12.0	17.1	11.7
	Conformer_bi	76.5	72.7	77.2	75.5	5.9	10.2	14.6	10.2
	CAs-Net (Ours)	1.8	10.2	15.8	9.3	4.6	9.2	14.3	9.4
METRO (−6 dB)	Squeezeformer	108.7	99.4	100.0	102.7	114.1	99.3	100.0	104.4
	Squeezeformer_bi	95.1	88.7	89.8	91.2	15.2	39.6	48.5	34.4
	Transformer	120.3	99.3	99.1	106.2	86.3	95.8	96.0	92.7
	EfficonformerV2	109.9	99.5	100.0	103.1	118.1	99.5	99.9	105.8
	EfficonformerV1	108.2	99.1	99.3	102.2	115.3	99.9	99.9	105.0
	Conformer	18.7	50.6	58.1	42.5	7.1	26.3	35.1	22.8
	Conformer_bi	100.1	95.5	96.2	97.3	11.9	34.5	43.1	29.8
	CAs-Net (Ours)	11.5	31.4	38.9	27.3	12.0	17.4	25.7	18.4

**Table 4:** Comparison in real-world environments.

Model	Train	Ug 7	Ug 9	Ug 16	Ug 7	Ug 9	Ug 16
		+BUS (−6 dB)			+BUS (−6 dB)		
	Test	Real1			Real2		
Transformer		116.0	13.4	23.5	118.5	46.4	31.0
EfficonformerV2		6.5	4.2	6.7	12.5	30.1	7.9
EfficonformerV1		12.7	7.3	5.9	18.5	34.3	12.5
Conformer		17.6	6.3	5.9	26.2	31.8	7.6
Conformer_bidecoder		89.3	6.0	5.5	91.2	35.2	7.2
CAs-Net (Ours)		5.6	2.6	2.5	10.7	7.0	5.9

**Table 5:** Statistical significance of WER improvement on Ug7 dataset at −6 dB SNR.

Experiment	Data	*p*-Value
Overall Comparison	Full Dataset	$5.9482\times 10^{-7}$
Random Sample 1	100 utterances	$3.8674\times 10^{-2}$
Random Sample 2	100 utterances	$2.1338\times 10^{-1}$
Random Sample 3	100 utterances	$9.7307\times 10^{-3}$
Random Sample 4	100 utterances	$8.0203\times 10^{-2}$
Random Sample 5	100 utterances	$1.8346\times 10^{-4}$
Random Sample 6	100 utterances	$4.4929\times 10^{-2}$
Random Sample 7	100 utterances	$2.7803\times 10^{-1}$
Random Sample 8	100 utterances	$2.5593\times 10^{-5}$
Random Sample 9	100 utterances	$1.4408\times 10^{-2}$
Random Sample 10	100 utterances	$4.3333\times 10^{-3}$

**Table 6:** Ablation results under BUS and METRO noise conditions at −6 dB SNR.

Noise	Model Variant	WER			
		Ug7	Ug8	Ug9	Ug16
BUS (−6 dB)	CAs-Net	1.75	1.50	2.51	4.56
	-MSDCM	1.96	2.15	3.05	4.59
	-O3	2.04	1.91	2.89	4.86
	-O2-O3	2.34	2.30	3.08	4.94
METRO (−6 dB)	CAs-Net	11.45	8.01	9.12	11.98
	-MSDCM	12.84	8.66	9.87	12.40
	-O3	12.37	8.37	9.55	12.06
	-O2-O3	14.71	9.11	10.24	12.77

**Table 7:** Ablation results under BUS and METRO noise conditions at −3 dB SNR.

Noise	Model Variant	WER			
		Ug7	Ug8	Ug9	Ug16
BUS (−3 dB)	CAs-Net	2.19	1.03	2.16	4.28
	-MSDCM	2.79	1.2	2.63	4.34
	-O3	2.87	1.97	2.71	4.31
	-O2-O3	2.94	2.28	2.98	4.41
METRO (−3 dB)	CAs-Net	10.03	1.57	3.30	5.26
	-MSDCM	10.26	1.65	3.71	5.78
	-O3	10.27	1.64	3.9	5.34
	-O2-O3	10.31	3.21	4.55	5.41

## Data Availability

The data that support the findings of this study are openly available in a public repository.
